# Medication Intake, Perceived Barriers, and Their Correlates Among Adults With Type 1 and Type 2 Diabetes: Results From Diabetes MILES – The Netherlands

**DOI:** 10.3389/fcdhc.2021.645609

**Published:** 2021-04-30

**Authors:** Stijn Hogervorst, Marce C. Adriaanse, Jacqueline G. Hugtenburg, Mariska Bot, Jane Speight, Frans Pouwer, Giesje Nefs

**Affiliations:** ^1^ Department of Health Sciences, Vrije Universiteit Amsterdam, Amsterdam, Netherlands; ^2^ Amsterdam Public Health Research Institute (APH), Amsterdam University Medical Centre, Amsterdam, Netherlands; ^3^ Department of Clinical Pharmacology and Pharmacy, Amsterdam University Medical Centres (UMC), Amsterdam, Netherlands; ^4^ Department of Psychiatry and the Amsterdam Public Health Institute, VU University Medical Center, Amsterdam, Netherlands; ^5^ School of Psychology, Deakin University, Geelong, VIC, Australia; ^6^ The Australian Centre for Behavioural Research in Diabetes, Diabetes Victoria, Melbourne, VIC, Australia; ^7^ Department of Psychology, University of Southern Denmark, Odense, Denmark; ^8^ Steno Diabetes Center Odense (SDCO), Odense, Denmark; ^9^ Center of Research on Psychological and Somatic Disorders (CoRPS), Department of Medical and Clinical Psychology, Tilburg University, Tilburg, Netherlands; ^10^ Department of Medical Psychology, Radboud University Medical Center, Radboud Institute for Health Sciences, Nijmegen, Netherlands; ^11^ Diabeter, National Treatment and Research Center for Children, Adolescents and Adults With Type 1 Diabetes, Rotterdam, Netherlands

**Keywords:** medication intake, type 1 diabetes, type 2 diabetes, national survey, association model

## Abstract

**Purpose:**

The purpose of this study is to investigate medication intake, perceived barriers and their correlates in adults with type 1 or type 2 diabetes.

**Methods:**

In this cross-sectional study, 3,383 Dutch adults with diabetes (42% type 1; 58% type 2) completed the 12-item ‘Adherence Starts with Knowledge’ questionnaire (ASK-12; total score range: 12-60) and reported socio-demographics, clinical and psychological characteristics and health behaviors. Univariable and multivariable logistic regression analyses were used.

**Results:**

Adults with type 1 diabetes had a slightly lower mean ASK-12 score (i.e. more optimal medication intake and fewer perceived barriers) than adults with non-insulin-treated type 2 diabetes. After adjustment for covariates, correlates with suboptimal intake and barriers were fewer severe hypoglycemic events and more depressive symptoms and diabetes-specific distress. In type 2 diabetes, correlates were longer diabetes duration, more depressive symptoms and diabetes-specific distress.

**Conclusions:**

Adults with type 1 diabetes showed slightly more optimal medication intake and fewer perceived barriers than adults with non-insulin treated type 2 diabetes. Correlates differed only slightly between diabetes types. The strong association with depressive symptoms and diabetes-specific distress in both diabetes types warrants attention, as improving these outcomes in some people with diabetes might indirectly improve medication intake.

## Introduction

The global prevalence of both type 1 and type 2 diabetes is at an all-time high while incidence and prevalence rates continue to increase (1, 2). Diabetes-related costs represent one of the highest expenditures in healthcare systems and can be attributed mainly to the high morbidity associated with diabetes (3–6).

The cornerstone of optimal glycemic outcomes in both type 1 and type 2 diabetes relies on diligent self-management, of which medication intake is a central element (7, 8). However, only 50% of adults with type 2 diabetes have an A1C of <7% (53 mmol/mol) (9). For type 1 diabetes, the rate varies between 20-60%, depending on country and age group (10). The difficulty in meeting these target values is often attributed to suboptimal medication intake (3). In adults with type 2 diabetes optimal medication intake ranges from 39% to 93% with large differences between studies, populations and measurement methods (11).

People have a myriad of reasons for not taking their medication as recommended, as illustrated by Kardas et al. who identified no less than 771 different factors in a review of systematic reviews (12). There is a vast body of research exploring medication intake and its correlates in adults with type 2 diabetes. Using a model developed by the World Health Organization (13), the most relevant factors can be categorized as person-related (e.g. younger age, low health literacy, being male, having depression), socio-economic (e.g. affordability of medication, less social support), diabetes-related (e.g. shorter diabetes duration), treatment-related (e.g. more complicated medication regimen, more side-effects, insulin use) and healthcare-related (e.g. patient-clinician communication, lack of time for adequate care, lack of integrated care) (14–16). Far less is known about factors influencing medication intake in adults with type 1 diabetes. They were found to have a two-fold higher risk of “medication errors” at hospital admission as compared with adults with type 2 diabetes, *defined by the authors as “unintentional medication discrepancies corrected by physicians” of very serious (potentially leading to life-threatening consequences) or serious (potentially causing harm or extended hospital stay) nature* (17). Of note, people with type 1 diabetes were more often admitted through the emergency department, more often had medication errors involving added medications and more medication errors per treatment compared to people with type 2 diabetes. Understanding the factors that influence medication intake will help clinicians and policy makers to provide better support to adults with diabetes in maintaining the medication intake necessary to achieve optimal glycemic control.

The characteristics of people with type 1 and type 2 diabetes differ on a number of important points. Around half of adults with type 1 diabetes have had the condition since childhood or adolescence whereas those with type 2 diabetes generally developed the condition later in life. In terms of etiology, type 1 diabetes is characterized by an absolute insulin deficiency resulting from an autoimmune process, whereas type 2 is characterized by insulin resistance and a relative insulin deficiency related to factors such as obesity, increasing age and genetic disposition (1). In line with this, the treatment approaches for both types are different. First, type 1 diabetes is always treated with insulin therapy (through injections or pump) and monitoring of glucose levels with a blood glucose meter or a continuous glucose monitoring system (CGM). Type 2 diabetes can often be treated with a combination of a diet and oral medication. When oral medication is no longer sufficient, insulin injection therapy can be added. These differences also translate into different diabetes self-management activities. For example, people with type 1 diabetes often apply complex treatment regimens including multiple daily insulin doses and carbohydrate counting. On the other hand people with type 2 diabetes do not always have to inject insulin, but cope more often with multi-morbidity and multi-pharmacy. Consequently, medication intake, perceived barriers and their correlates might also differ between diabetes types.

A better understanding of medication intake, perceived barriers and their correlates will help clinicians, policy makers and researchers to tailor treatment and interventions to the needs of individual persons with diabetes from both target populations. Therefore, the aims of the present study were: (a) to investigate (self-reported) medication intake and perceived barriers in adults with type 1 and type 2 diabetes; and (b) to identify socio-demographic, psychological and clinical characteristics, and health behaviors that influence medication intake and perceived barriers.

## Materials and Methods

### Participants and Procedure

Data were extracted from Diabetes MILES (Management and Impact for Long-term Empowerment and Success) – The Netherlands study. The MILES study is a national, online, cross-sectional observational study designed to examine the psychosocial aspects of living with diabetes. The study’s rationale, design and procedure has been described in detail elsewhere (18). From September to October 2011, Dutch adults (aged ≥19 years, no upper age limit) with self-reported diabetes of any type were offered the opportunity through several media channels to participate in an online survey. The study protocol was approved by the Psychological Research Ethics Committee of Tilburg University, The Netherlands (EC-2011 5). Written informed consent was obtained digitally from all participants.

The subsample in the present study (N=3,383) includes all participants with self-reported type 1 or type 2 diabetes who completed the Adherence Starts with Knowledge 12 (ASK-12) questionnaire and who indicated that they are prescribed medication for their diabetes (either insulin pump or injection, GLP-1 injection, blood glucose lowering tablets or a combination of those) (19).

### Medication Intake

Medication intake and perceived barriers were assessed using the ASK-12 (19). The ASK-12 questionnaire has demonstrated validity and internal reliability consistency in people with type 2 diabetes and was validated both by making use of another questionnaire (the Morisky Medication Adherence Scale) and by making use of filled medication prescription data from pharmacies (19). In the current study Cronbach’s alpha indicated that the ASK-12 is reliable for both adults with type 1 and type 2 diabetes (Cronbach’s alpha = 0.66 and 0.68 respectively). Additionally, lower ASK-12 total scores (i.e. more favorable medication intake and less perceived barriers) are associated with lower HbA1c in both type 1 diabetes (Logistic regression analysis, n = 1172, B = 0.039, p-value = 0.001) and type 2 diabetes with insulin (logistic regression analysis, n = 718, B = 0.031, p-value = 0.026) and type 2 diabetes without insulin (logistic regression analysis, n = 568, B = 0.033, p-value = 0.029).

The questionnaire includes twelve questions, rated from 1 (totally disagree) to 5 (totally agree) for the first seven questions and from 1 (in the past week) to 5 (never) for the final 5 questions. Responses are summed to generate a total score (range: 12-60). Three subscale scores can also be derived from the ASK-12: behavior (5 items, score range 5-25, e.g. ‘*Have you not had medicine with you when it was time to take it?*’), health beliefs (4 items, score range 4-20, e.g. *‘I feel confident that each of my medicines will help me*’) and inconvenience/forgetfulness (3 items, score range 3-15, e.g. ‘*Sometimes I simply forget to take my medications’*). Higher ASK-12 total and subscale scores indicate suboptimal medication intake and more perceived barriers (i.e. more problematic beliefs and greater inconvenience/forgetfulness).

### Psychological Characteristics

Symptoms of depression and anxiety during the past two weeks were measured using the validated 9-item Patient Health Questionnaire (PHQ-9; total score range: 0–27) (20) and 7-item General Anxiety Disorder questionnaire (GAD-7; total score range: 0–21) (21). For both measures, higher scores indicate higher levels of symptoms, and scores ≥10 indicate elevated symptoms of depression or anxiety. Diabetes-specific emotional distress was assessed with the validated 20-item Problem Areas in Diabetes scale (PAID; total score range: 0–100), in which higher scores indicate greater severity of diabetes-specific emotional distress (22).

### Socio-demographics, Clinical Characteristics and Health Behaviors

The Diabetes MILES – The Netherlands survey included several items on socio-demographic characteristics (i.e., sex, age, ethnic background, educational level, marital status and work status). Participants also self-reported their height/weight (enabling calculation of their Body Mass Index (BMI)), alcohol use (eight categories from 0 to ≥36 glasses per week), being a daily smoker, diabetes duration, current diabetes treatment, most recent A1C (continuous variable and dichotomized using the cut-off of ≥7%/53 mmol/mol to indicate a sub-optimal glycemic outcome), the number of severe hypoglycemic events in the past year (defined as a low blood glucose level requiring assistance from another person for recovery), conditions that might be vascular complications of diabetes (e.g. myocardial infarction, stroke, peripheral arterial disease, nephropathy, retinopathy, neuropathy and/or foot ulcers) and somatic comorbid conditions (e.g. hypertension, high cholesterol, chronic heart failure, asthma/chronic obstructive pulmonary disease (COPD) and rheumatic disorders/joint problems). The number of medications for comorbidity was calculated by making an aggregation variable of medication taken by participants for 32 different diseases, including diabetes-related complications.

### Statistical Analyses

Socio-demographics, clinical and psychological characteristics and health behaviors were described as mean ± standard deviation (SD) for continuous variables and % (*n*/*N*) for categorical variables, stratified by diabetes type. First, differences in the ASK-12 total score and the three ASK-12 subscales were compared between adults with type 1 and insulin-treated and non-insulin-treated type 2 diabetes using independent sample t-tests. Sample size adjusted effect sizes (Cohen’s *d* statistic) were calculated.

Subsequently, the association of socio-demographics, psychological and clinical characteristics and health behaviors with less favorable medication intake and more perceived barriers was analyzed separately by diabetes type. A ‘suboptimal medication intake and more perceived barriers’ group was created based on the ASK-12 total score, by taking the least positive scoring quartile (i.e. the 25% with the highest score) for both types of diabetes. Two separate univariable logistic regression analyses were used to determine risk markers in adults with type 1 diabetes and type 2 diabetes respectively. To avoid overfitting (23), we selected the following thirteen potential correlates *a priori* based upon clinical considerations, literature review and availability: sex, age, education level, having a partner, diabetes duration, number of medications for comorbid conditions, number of visits with a clinician in the past year, frequency of severe hypoglycemic events in the past year, diabetes-specific distress, anxiety symptoms, depressive symptoms, alcohol use and smoking behavior. Thereafter, two multiple logistic regression analyses were conducted to create an association model with correlates that contribute to suboptimal medication intake and more perceived barriers in adults with type 1 and type 2 diabetes, respectively. All factors that showed a *p*-value <0.10 in the univariate analyses were added to the multivariable association model. Analyses were performed using SPSS Statistics version 24 (IBM, Somers, NY, USA). A *p*-value <0.05 was considered to be statistically significant.

## Results

### Sample Characteristics

The total sample consisted of 3,383 adults with diabetes, of whom 42% (n=1,422) had type 1 diabetes and 58% (n=1,961) had type 2 diabetes. **Table 1** shows the socio-demographics, psychological and clinical characteristics and health behaviors of these groups.

**Table 1:** Participants’ socio-demographics, psychological and clinical characteristics and health behaviors, stratified by diabetes type (N=3,383).

	N Missing	Type 1 diabetes(n=1,422)	Type 2 diabetes(n=1,961)
*SOCIO-DEMOGRAPHICS*			
**Female sex**	1	61 (860/1421)	49 (952/1961)
**Age, years**	49	47.6 (14.7)	61.7 (10.1)
**(non-Dutch) ethnic minority**	0	2 (31/1422)	3 (51/1961)
**Low educational level**	8	19 (264/1421)	32 (630/1954)
**Being single**	0	20 (278/1422)	21 (403/1961)
**Paid work**	6	63 (897/1420)	36 (708/1957)
**Body Mass Index, kg/cm^2^**	36	25.5 (4.7)	29.8 (5.9)
*CLINICAL CHARACTERISTICS*			
**Diabetes duration, years**	3	23.5 (14.6)	11.1 (8.1)
**Primary diabetes treatment**	18		
- Insulin pump		49 (693/1422)	6 (116/1943)
- Insulin injections		51 (729/1422)	50 (933/1943)
- GLP-1 injections		0	2 (35/1943)
- Blood glucose lowering tablets		0	42 (787/1943)
**Most recent A1C, mmol/mol**	924	58 (12)	54 (12)
**Most recent A1C, %**	924	7,5 (3.2)	7,1 (3.2)
**Suboptimal A1C (≥7%, ≥53 mmol/mol)**	924	70 (826/1172)	50 (645/1287)
**N° severe hypoglycemic events in past year**	22	1.4 (6.4)	0.4 (2.9)
**N° medications for comorbid conditions**	1	1.3 (1.5)	2.1 (1.7)
**N° appoints with clinicians in past year**	26	19.4 (20.7)	20.2 (22.5)
**At least one diabetes complication**	0	32 (448/1422)	33 (641/1961)
**Microvascular**			
- Retinopathy	0	19 (263/1422)	6 (126/1961)
- Neuropathy	0	17 (237/1422)	18 (350/1961)
- Nephropathy	0	5 (64/1422)	4 (79/1961)
- Foot condition due to diabetes	0	4 (63/1422)	5 (106/1961)
**Macrovascular**			
- Myocardial infarction	0	4 (52/1422)	7 (130/1961)
- Stroke	0	1 (18/1422)	3 (54/1961)
- Peripheral arterial disease	0	3 (39/1422)	6 (116/1961)
**Other co-morbid chronic conditions**			
- Hypertension	0	25 (353/1422)	51 (993/1961)
- High cholesterol	0	23 (328/1422)	45 (877/1961)
- Chronic heart failure	0	1 (18/1422)	3 (54/1961)
- Asthma/COPD	0	8 (109/1422)	12 (231/1961)
- Rheumatic disorders/joint problems	0	12 (169/1422)	20 (387/1961)
*PSYCHOLOGICAL CHARACTERISTICS*			
**Anxiety symptoms (GAD7 total score)**	194	3.1 (3.6)	2.7 (3.6)
**Depressive symptoms (PHQ9 total score)**	188	4.3 (4.7)	4.3 (4.6)
**Diabetes-specific distress (PAID total score)**	174	22.3 (19.3)	19.5 (19.1)
*HEALTH BEHAVIOURS*			
**Daily smoker**	115	11 (151/1376)	8 (155/1892)
**Alcohol >14 glasses/week**	110	9 (125/1380)	6 (118/1893)

Data are presented as mean (± SD) or % (n/N).

### Medication Intake by Subgroup

Self-reported medication intake and perceived barriers (ASK-12 total and subscale scores) are shown for diabetes type 1, insulin-treated diabetes type 2 and non-insulin-treated diabetes type 2 in **Table 2**. Adults with type 1 diabetes reported slightly more optimal medication intake and fewer perceived barriers (i.e. lower total score ASK-12) than adults with non-insulin-treated type 2 diabetes (mean score: 21.2 ± 5.6 vs. 22.0 ± 6.0 respectively, *p*=0.005, Cohen’s *d*=0.14). No significant difference was found between people with type 1 diabetes and people with insulin-treated type 2 diabetes. With respect to the ASK-12 subscales, adults with type 1 diabetes reported more optimal medication intake behavior than adults with insulin-treated type 2 diabetes (7.2 ± 2.6 vs. 7.5 ± 2.9 resp., *p*=0.022, Cohen’s *d*=0.11) and than adults with non-insulin-treated type 2 diabetes (7.2 ± 2.6 vs. 7.7 ± 2.8 resp., *p*=<0,001, Cohen’s *d*=0.19). No significant differences were found between groups on the subscales health beliefs and inconvenience/forgetfulness.

**Table 2:** Medication intake and perceived barriers (mean ± SD) stratified by diabetes type and insulin use. Lower scores imply more optimal medication intake and fewer perceived barriers, based on the ASK-12 and three different subscales of the ASK-12^19^ (N = 3,383).

	Type 1 (n=1,422)	Type 2 insulin-treated (n=1,049)	Type 2 non-insulin treated (n=822)	Cohen’s d*	Significance
**Medication intake and perceived barriers** (12-60)	21.2 (5.6)	21.7 (5.8)	22.0 (6.0)	0.14	B
**Behavior** (5-25)	7.2 (2.6)	7.5 (2.9)	7.7 (2.8)	0.11, 0.19	A, B
**Health beliefs** (4-20)	8.3 (2.9)	8.6 (3.0)	8.6 (3.1)		NS
**Inconvenience/forgetfulness** (3-15)	5.7 (2.4)	5.6 (2.4)	5.8 (2.4)		NS

A = Significant difference between type 1 and insulin-treated type 2 at the p < 0,05 level, based on an ANOVA with post-hoc Bonferroni.

B = Significant difference between type 1 and non-insulin-treated type 2 at the p < 0,05 level, based on an ANOVA with post-hoc Bonferroni.

C = Significant difference between type 2 with insulin and non-insulin-treated type 2 at the p < 0,05 level, based on an ANOVA with post-hoc Bonferroni.

NS = no significant difference found between any of the groups at the p < 0,05 level, based on an ANOVA with post-hoc Bonferroni.

*Cohen’s d is only shown when significant differences have been found between groups.

### Correlates of Medication Intake and Perceived Barriers

**Table 3** shows correlates with less optimal medication intake and more perceived barriers (subgroups based on the dichotomized ASK-12 total score) in adults with type 1 and type 2 diabetes, based on univariable and multivariable regression analyses.

**Table 3:** Correlates with less optimal medication intake and perceived barriers, defined as the quartile with the highest ASK-12 total score (type 1 diabetes: n = 346/type 2 diabetes: n = 492), based on univariable and multivariable regression analyses, stratified by diabetes type (N=3,077).

	Type 1 diabetes (n=1,315)		Type 2 diabetes (n=1,760)	
	Univariable^a^	Multivariable^b^	Univariable^a^	Multivariable^b^
	OR (95% CI)	OR (95% CI)	OR (95% CI)	OR (95% CI)
*Socio-demographic characteristics*				
**Female sex**	1.12 (0.88 – 1.44)		1.32 (1.08 – 1.61)**	1.02 (0.81 – 1.28)
**Age, years**	0.99 (0.98 – 0.99)**	0.99 (0.98 – 1.00)	0.98 (0.97 – 0. 98)***	0.99 (0.98 – 1.00)
**Higher education level**	1.07 (0.91 – 1.25)		1.06 (0.93 – 1. 19)	
**Having a partner**	0.84 (0.63 – 1.13)		0.72 (0.57 – 0.92)**	0.84 (0.64 – 1.10)
*Clinical characteristics*				
**Severe hypoglycemic events past year**	0.99 (0.96 – 1.01)	0.97 (0.93 – 1.00)*	0.97 (0.93 – 1.02)	0.95 (0.90 – 1.01)
**Diabetes duration, years**	0.99 (0.98 – 1.00)*	0.97 (0.99 – 1.05)	0.97 (0.96 – 0.99)***	0.98 (0.97 – 1.00)*
**Number of appointments clinicians past year**	1.01 (1.00 – 1.01)**	1.00 (1.00 – 1.01)	1.00 (1.00 – 1.01)	1.00 (0.99 – 1.00)
Number of medications for comorbidity^f^	1.07 (0.99 – 1.15)	1.07 (0.97 – 1.17)	1.03 (0.97 – 1.10)	
*Health behaviors*				
**Daily smoker**	1.38 (0.96 – 1.99)		1.04 (0.71 – 1.50)	
**Alcohol use**	1.01 (0.92 – 1.11)		0.97 (0.92 – 1.07)	
*Psychological characteristics*				
**Anxiety symptoms** (GAD7 total score)^c^	1.12 (1.08 – 1.15)***	0.97 (0.92 – 1.02)	1.14 (1.11 – 1.17)***	1.01 (0.97 – 1.06)
**Depressive symptoms** (PHQ9 total score)^d^	1.12 (1.09 – 1.15)***	1.06 (1.01 – 1.11)**	1.13 (1.10 – 1.15)***	1.07 (1.03 – 1.11)***
**Diabetes-specific distress** (PAID total score)^e^	1.04 (1.04 – 1.05)***	1.03 (1.02 – 1.04)***	1.03 (1.03 – 1.04)***	1.02 (1.01 – 1.03)***

a Logistic regression analyses were used to calculate odds ratio (OR) and 95% confidence interval (CIs) for each risk factor independently.

b An association model was calculated adding all variables that contributed to the univariate model (p > 0,10) to the multivariate analysis.

c Generalized Anxiety Disorder questionnaire (range 0-21) in which a higher score implies more symptoms of anxiety^21^.

d Patient Health Questionnaire (range 0-28) in which a higher score implies more depressive symptoms^20^.

e Problem Areas in Diabetes Questionnaire (range 0-100) in which a higher score implies a more diabetes-specific distress^22^.

f Aggregation variable of medication for 32 different chronic diseases and diabetes complications.

*P-value <0,05/** P-Value <0,01/*** P-Value <0,00.

In the univariable analyses for adults with type 1 diabetes, less optimal medication intake and more perceived barriers were correlated with younger age (*p*=0.001), shorter diabetes duration (*p*=0.023), more appointments with clinicians in the past year (*p*<0.001), more anxiety symptoms (*p*<0.001), more depressive symptoms (*p*<0.001) and more diabetes-specific distress (*p*<0.001). In the multivariable analysis, less optimal medication intake and more perceived barriers were correlated with fewer severe hypoglycemic events in the past year (*p*=0.042), more depressive symptoms (*p*=0.012) and more diabetes-specific distress (*p*<0.001).

In the univariable analyses for adults with type 2 diabetes, less optimal medication intake and more perceived barriers were correlated with being female (*p*=0.007), younger age (*p*<0.001), not having a partner (*p*=0.008), shorter duration of diabetes (p<0.001), more anxiety symptoms (*p*<0.001), more depressive symptoms (*p*<0.001) and higher diabetes-specific distress (*p*<0.001). In the multivariate analysis, less optimal medication intake and more perceived barriers were correlated with shorter duration of diabetes (*p*=0.047), more depressive symptoms (*p*=0.001) and more diabetes-specific distress (*p*<0.001).

## Discussion

### Key Findings

The present study showed that adults with type 1 diabetes had slightly more optimal medication intake and fewer perceived barriers (i.e. lower ASK-12 total score) than adults with non-insulin-treated type 2 diabetes. Additionally, they had slightly more optimal scores on the behavior subscales than adults with both insulin-treated and non-insulin-treated type 2 diabetes, but did not differ with respect to the subscales health beliefs or inconvenience/forgetfulness. Correlates of less optimal medication intake and more perceived barriers in type 1 diabetes were fewer severe hypoglycemic events in the past year, higher depressive symptoms and higher diabetes-specific distress. Correlates of less optimal medication intake and more perceived barriers in type 2 diabetes were a shorter duration of diabetes, more depressive symptoms and more diabetes-specific distress.

### Interpretations and Comparison to Literature

Adults with type 1 diabetes showed slightly more optimal medication intake and fewer perceived barriers than adults with non-insulin-treated type 2 diabetes and showed slightly more optimal medication intake behavior than adults with both insulin-treated and non-insulin-treated type 2 diabetes. To our knowledge, there has been no previous research comparing levels of medication intake and perceived barriers between adults with type 1 and type 2 diabetes. However, our findings need to be interpreted with care, as the absolute difference on the ASK-12 total score was very small and there is no clinically relevant cut-off point on the ASK-12 for less optimal medication intake and more perceived barriers in diabetes patients (19). Moreover, Diabetes MILES – The Netherlands is an online, self-report survey, and probably less representative than a population-based study. The largest difference was found on the medication intake behavior subscale, which describes the degree to which a person intentionally misses a dose for various reasons.

The slightly more optimal medication intake and fewer perceived barriers in people with type 1 diabetes compared to people with type 2 diabetes could be explained by differences in pathophysiology. Specifically, people with type 1 diabetes experience more immediate risks of ketoacidosis when not administering insulin, whereas the direct consequences are less dire when people with type 2 diabetes miss multiple doses of glucose lowering tablets. Correlates of less optimal medication intake and more perceived barriers in type 1 diabetes were fewer severe hypoglycemic events in the past year, higher depressive symptoms and higher diabetes-specific distress. With regard to correlates of medication intake and perceived barriers in adults with type 1 diabetes, the literature is unclear. Main reasons are that most studies look at glycemic outcomes or HbA_1c_ as outcome rather than medication intake. Additionally, most studies differ in terms of determinants included. Our findings for the correlates of less optimal medication intake and more perceived barriers in adults with type 2 diabetes are consistent with previous research showing the role of higher depressive symptoms (14–16). Clearly, in both types of diabetes many different factors play a role in medication intake behavior and perceived barriers. Therefore, clinicians need to openly and constructively discuss the various reasons underlying an individual’s suboptimal medication intake. Furthermore, the associations with severe hypoglycemia (in type 1 diabetes) and diabetes duration (in type 2 diabetes) warrant attention, as these factors might be indicative of worse medication intake and more perceived barriers.

Another important finding from this study is the strong association of medication intake and perceived barriers with two psychological factors (depressive symptoms and diabetes-specific distress), which was implicated both in adults with type 1 and type 2 diabetes. The relationship between diabetes and depressive symptoms is well-established and appears to be bi-directional. As compared to adults without diabetes, adults with diabetes have a two-to-threefold increased risk of depression (24). Additionally, adults with depression have a 1.76 odds of suboptimal medication intake compared to adults without depression (25).People with T2DM and co-morbid depression show less optimal medication intake behavior, as well as more frequent hyperglycemia (26, 27). The association between depressive symptoms and sub-optimal medication intake is possibly caused by various aspects of depression such as a lack of motivation, a lack of energy, difficulties in making decisions and a lower self-esteem. Comparable results have been found for diabetes-specific distress, since higher diabetes-specific distress was associated with less optimal medication intake, mediated through perceived control and self-efficacy (28). Therefore, clinicians need to especially consider psychological factors as potential barriers for optimal medication intake. Additionally, clinicians might consider making use of the wide range of interventions developed specifically to target diabetes distress or depression in people with diabetes, as they have been shown to lower both depressive symptoms, diabetes distress and HbA1c (29, 30). However, we need to be cautious with respect to glycemic effects, as improvement of the general medical condition including glycemic control is likely to require simultaneous attention to both conditions.”

### Strengths and Limitations

The strengths of this study include the use of a large dataset including adults with type 1 diabetes or type 2 diabetes, the extensive number of variables available in this dataset and the use of both univariable and multivariable analyses. Another strength is the use of a validated and reliable questionnaire to measure medication use (19), as well as depressive symptoms and diabetes-specific distress. However, analyses in this study were focused solely on this single self-report measurement of medication intake and perceived barriers, which may be subject to some social desirability bias. Additionally, the results from this study do not include any objective data on medication intake. The suitability of the ASK-12 as a measure of medication intake and perceived barriers for people with type 1 diabetes can be strengthened. Future rigorous psychometric testing of the instrument in this group is recommended. The validity of the results could therefore be improved by making use of an objective medication intake measurement such as medication event monitoring systems (MEMS) (31). MEMS, however, are an expensive data collection method, which is not feasible to implement in an extensive project such as the MILES study.

Additionally, the Diabetes MILES – Netherlands sample of adults with type 1 diabetes and type 2 diabetes in the dataset is not fully representative of adults with type 1 or type 2 diabetes in the Netherlands. For example, adults with type 2 diabetes who do not use insulin and adults from ethnic minority groups were underrepresented (18). Also people with lower HbA_1c_ and emotional distress were overrepresented in the present sample (32, 33). Moreover, the subpopulation evaluated in the present study showed higher medication intake scores and fewer perceived barriers as compared to the diabetes population participating in the ASK-12 publication study (19).

Diabetes MILES – the Netherlands aimed to measure life with diabetes across diabetes types. Comparisons between type 1 and type 2 diabetes are possible because the same instruments were used to measure constructs in both conditions. As a consequence, sometimes more condition-specific and detailed information is missing. For example, the ASK-12 questionnaire has not been specifically validated in people with type 1 diabetes, and detailed information on the insulin regimen is lacking.

Finally, this is a cross-sectional observational study so we cannot infer causality from these findings.

## Conclusions

In conclusion, adults with type 1 diabetes show slightly more optimal medication intake and fewer perceived barriers than adults with non-insulin-treated type 2 diabetes and more optimal medication intake behavior than adults with both insulin-treated and non-insulin-treated type 2 diabetes. However, these differences were not found on all subscales and absolute differences between groups are minimal. Correlates of less optimal medication intake and more perceived barriers in type 1 diabetes are fewer severe hypoglycemic events in the past year, higher depressive symptoms and higher diabetes-specific distress. Factors associated with less optimal medication intake and more perceived barriers in type 2 diabetes are a shorter duration of diabetes, higher depressive symptoms and higher diabetes-specific distress.

### Recommendations for Practice

These insights suggest practical ways in which clinicians can better support people with diabetes, tailoring their interventions to the specific reason(s) for suboptimal medication intake and unmet needs of the individual with diabetes. Especially the strong association with depressive symptoms and diabetes-specific distress in both diabetes types warrants attention, as improving these outcomes in some persons with diabetes might indirectly improve medication intake.

## Data Availability Statement

The raw data supporting the conclusions of this article will be made available by the authors, without undue reservation.

## Ethics Statement

The study protocol was approved by the Psychological Research Ethics Committee of Tilburg University, The Netherlands (EC-2011 5). The patients/participants provided their written informed consent to participate in this study.

## Author Contributions

All authors contributed to the conception and design of the study, analysis, and interpretation of the data. SH, MA, and JH drafted the first version of the manuscript. All authors (SH, MA, JH, MB, JS, FP, GN) critically revised the manuscript for important intellectual content. All authors contributed to the article and approved the submitted version.

## Funding 

This study was supported by the Prof. dr. J. Terpstra Young Investigator Award 2010 from the Dutch Association for Diabetes Research (Nederlandse Vereniging voor Diabetes Onderzoek)/Lilly Diabetes to GN. The funding source had no role in the design, data collection, analysis or interpretation of the study, or in the decision to submit the manuscript for publication. JS is supported by core funding to the Australian Centre for Behavioural Research in Diabetes, derived from the collaboration between Diabetes Victoria and Deakin University.

## Conflict of Interest

The authors declare that the research was conducted in the absence of any commercial or financial relationships that could be construed as a potential conflict of interest.
