# Supplementary material for: Medication Intake, Perceived Barriers, and Their Correlates Among Adults With Type 1 and Type 2 Diabetes: Results From Diabetes MILES – The Netherlands
Source: Front Clin Diabetes Healthc. 2021 Apr 30;2:645609. doi: 10.3389/fcdhc.2021.645609 (PMC10012124; doi:10.3389/fcdhc.2021.645609)
Supplement: Supplementary file 1 [file DataSheet_1.docx]

Manuscript ID: 645609

Article Title: Medication intake, perceived barriers and their correlates among adults with type 1 and type 2 diabetes: Results from Diabetes MILES – The Netherlands

Journal Name: Frontiers in Clinical Diabetes and Healthcare, section Diabetes Self-Management

| **Supplementary table 1.** Correlation matrix for HbA_1c_ and different questionnaires in people with type 1 diabetes (n = 1422) | | | | | |
| --- | --- | --- | --- | --- | --- |
|  | ASK-12 total score | PAID total score | PHQ9 total score | GAD7 total score | HbA_1c_ |
| ASK-12 total score | 1 | 0.39* | 0.34* | 0.27* | 0.18* |
| PAID total score |  | 1 | 0.61* | 0.56* | 0.22* |
| PHQ9 total score |  |  | 1 | 0.77* | 0.16* |
| GAD7 total score |  |  |  | 1 | 0.11* |
| HbA_1c_ |  |  |  |  | 1 |

*Correlation is significant at the 0.001 level (2-tailed)

| **Supplementary table 2**. Correlation matrix for HbA_1c_ and different questionnaires in people with type 2 diabetes without insulin (n = 910) | | | | | |
| --- | --- | --- | --- | --- | --- |
|  | ASK-12 total score | PAID total score | PHQ9 total score | GAD7 total score | HbA_1c_ |
| ASK-12 total score | 1 | 0.38* | 0.33* | 0.29* | 0.11* |
| PAID total score |  | 1* | 0.59* | 0.58* | 0.28* |
| PHQ9 total score |  |  | 1 | 0.78* | 0.16* |
| GAD7 total score |  |  |  | 1 | 0.16* |
| HbA_1c_ |  |  |  |  | 1 |

*Correlation is significant at the 0.001 level (2-tailed)

| **Supplementary table 3.** Correlation matrix for HbA_1c_ and different questionnaires in people with type 2 diabetes with insulin (n = 1049) | | | | | |
| --- | --- | --- | --- | --- | --- |
|  | ASK-12 total score | PAID total score | PHQ9 total score | GAD7 total score | HbA_1c_ |
| ASK-12 total score | 1 | 0.29* | 0.28* | 0.19* | 0.11* |
| PAID total score |  | 1 | 0.63* | 0.58 | 0.20* |
| PHQ9 total score |  |  | 1 | 0.78* | 0.10* |
| GAD7 total score |  |  |  | 1 | 0.09* |
| HbA_1c_ |  |  |  |  | 1 |

*Correlation is significant at the 0.001 level (2-tailed)

| **Alternative Table 3.** Correlates of medication intake and perceived barriers (ASK-12 total scores) plus variance explained (R^2^), based on univariable and multivariable linear regression analyses, stratified by diabetes type (N=3,077) | | | | | | | | | |
| --- | --- | --- | --- | --- | --- | --- | --- | --- | --- |
|  | **Type 1 diabetes (n=1,315)** | | | | **Type 2 diabetes (n=1,760)** | | | | |
|  | **Univariable^b^** | | **Multivariable^c^** | | **Univariable^b^** | | **Multivariable^c^** | | |
|  | **B (95% CI)** | **R^2^** | **B (95% CI)** |  | **B (95% CI)** | **R^2^** | **B (95% CI)** | |  |
| *Socio-demographic characteristics* |  |  |  |  |  |  |  |  | |
| **Female sex** | 0.56 (-0.04 – 1.15) | 0.002 |  |  | -0.09 (-0.12 – -0.06)** | 0.022 |  |  | |
| **Age, years** | -0.04 (-0.06 – -0.02)*** | 0.010 |  |  | 0.62 (0.10 – 1.14)*** | 0.003 | -0.04 (-0.06 – -0.01)** |  | |
| **Higher education level** | 0.02 (-0.37 – 0.40) | 0.000 |  |  | 0.20 (-0.12 – 0.52) | 0.001 | 0.36 (0.42 – 0.68)* |  | |
| **Having a partner** | -0.88 (0.63 – 1.13)* | 0.004 |  |  | -1.30 (-1.93 – -0.65)** | 0.008 | -0.64 (-1.27 – -0.01)* |  | |
| *Clinical characteristics* |  |  |  |  |  |  |  |  | |
| **Severe hypoglycaemic events past year** | -0.03 (-0.07 – 0.02) | 0.001 | -0.07 (-0.11 – -0.02)* |  | -0.04 (-0.13 – 0.05) | 0.000 | 0.95 (0.90 – 1.01) |  | |
| **Diabetes duration, years** | -0.03 (-0.52 – -0.01)** | 0.007 | -0.03 (-0.05 – 0.01)* |  | -0.06 (-0.09 – -0.03)*** | 0.007 | 0.98 (0.97 – 1.00)* |  | |
| **Number of appointments clinicians past year** | 0.03 (0.02 – 0,04)*** | 0.016 | 0.01 (-0.01 – 0.02) |  | 0.01 (-0.01 – 0.01) | 0.001 | -0.01 (-0.02 – 0.01) |  | |
| **Number of medications for comorbidity^g^** | 0.26 (0.07 – 0.45)** | 0.005 | 0.19 (-0.01 – 0.38) |  | 0.19 (0.04 – 0.35)* | 0.003 |  |  | |
| *Health behaviours* |  |  |  |  |  |  |  |  | |
| **Daily smoker** | 0.93 (-0.02 – 1.87) | 0.003 |  |  | 0.06 (-0.90 – 1.02) | 0.000 |  |  | |
| **Alcohol use** | 0.03 (-0.19 – 0.25) | 0.000 |  |  | -0.03 (-0.02 – 0.17) | 0.000 | 0.20 (-0.01 – 0.41) |  | |
| *Psychological characteristics* |  |  |  |  |  |  |  |  | |
| **Anxiety symptoms** (GAD7 total score)^d^ | 0.41 (0.33 – 0.48)*** | 0.074 |  |  | 0.39 (0.31 – 0.46)*** | 0.058 |  |  | |
| **Depressive symptoms** (PHQ9 total score)^e^ | 0.41 (0.35 – 0.47)*** | 0.116 | 0.20 (0.12 – 0.27)*** |  | 0.38 (0.32 – 0.43)*** | 0.090 | 0.22 (0.15 – 0.29)*** |  | |
| **Diabetes-specific distress** (PAID total score)^f^ | 0.11 (0.10 – 0.13)*** | 0.154 | 0.08 (0.07 – 0.10)*** |  | 0.10 (0.09 – 0.11)*** | 0.107 | 0.07 (0.05 – 0.09)*** |  | |

^a^ Lineair regression analyses were used to calculate regression coefficients (B) and 95% confidence interval (CIs) for each risk factor independently.
^b^ An association model was calculated adding all variables that contributed to the univariate model (p > 0,10) to the multivariate analysis.
^c^ Generalized Anxiety Disorder questionnaire (range 0-21) in which a higher score implies more symptoms of anxiety^21^.
^d^ Patient Health Questionnaire (range 0-28) in which a higher score implies more depressive symptoms^20^. ^e^ Problem Areas in Diabetes Questionnaire (range 0-100) in which a higher score implies a more diabetes-specific distress^22^.
^f^ Aggregation variable of medication for 32 different chronic diseases and diabetes complications.
* *P*-value <0,05 / ** *P*-Value <0,01 / *** *P*-Value <0,00

variance explained by multilevel regression model for type 1 diabetes: R^2^ = 0.191

variance explained by multilevel regression model for type 2 diabetes: R^2^ = 0.132
